# High prevalence of past hepatitis B virus infection in diffuse large B cell lymphoma: a retrospective study from Italy

**DOI:** 10.1007/s00277-023-05412-1

**Published:** 2023-08-31

**Authors:** Marcella Visentini, Andrea Pica, Giancarlo D’Ippolito, Eleonora Sculco, Francesca La Gualana, Laura Gragnani, Marzia Miglionico, Cesare Mazzaro, Massimo Fiorilli, Stefania Basili, Maurizio Martelli, Alice Di Rocco, Milvia Casato, Giuseppe Gentile, Alessandro Pulsoni

**Affiliations:** 1 Division of Clinical Immunology, Department of Translational and Precision Medicine, Sapienza University of Rome, Rome, Italy; 2 Department of Translational Research & NTMS, University of Pisa, Pisa, Italy; 3 Unit of Clinical of Experimental Onco-Haematology, IRCCS Centro di Riferimento Oncologico (CRO), Aviano, Pordenone, Italy; 4 Division of Hematology, Department of Translational and Precision Medicine, Sapienza University of Rome, Rome, Italy

**Keywords:** Hepatitis B virus, Past infection, Chronic infection, Diffuse large B-cell lymphoma

## Abstract

Studies from high endemic areas, mostly China, indicate that surface antigen positive (HBsAg^pos^) chronic hepatitis B virus (HBV) infection is associated with an increased risk of developing diffuse large B-cell lymphoma (DLBCL), whereas studies in low endemic areas have provided conflicting results. Past infection, serologically defined by negative HBsAg and positive anti-core antibody (HBsAg^neg^HBcAb^pos^), has also been suggested to increase the risk of B-cell non-Hodgkin’s lymphoma (NHL) in high endemic areas. We retrospectively reviewed unselected clinical records of 253 patients with DLBCL (54% male, aged 60.3 ± 14.6 years at diagnosis) and 694 patients with different types of indolent B-cell NHL (46% male, aged 61.7 ± 12.8 years). Patients were seen at a single center in Italy between 2001 and 2022 and HBV serological status (HBsAg, HBsAb, HBcAb, HBeAg, HBeAb, and HBV DNA) was analyzed through enzyme-linked immunosorbent assays and molecular assays; patients infected with hepatitis C virus or human immunodeficiency virus were excluded. We used an unconditional multiple logistic regression model including as matching variables gender, age at diagnosis, immigrant status, and HBV serological status. Patients with DLBCL had, compared to indolent NHL, a higher prevalence of HBsAg^pos^ active infection (odds ratio (OR) 2.8, 95% confidence interval (95% CI) 1.2–6.3, *p* = 0.014). Strikingly, patients with DLBCL had also a significantly higher prevalence of past infection (OR 2.4, 95% CI 1.5–4.0, *p* = 0.0006). Male gender was associated with increased risk of DLBCL independently of the HBV serological status. These findings suggest that both past and active HBV infection may increase the risk of DLBCL in a low endemic area. Our study needs confirmation by studies in areas or populations with different rates of chronic or past HBV infection.

## Introduction

Hepatitis B virus (HBV) is a hepatotropic DNA virus but also has the capacity to infect and replicate in lymphocytes and, likely because of it, to increase the risk of developing non-Hodgkin lymphoma (NHL) especially of B-cell type [1, 2]. Enhanced mutagenesis in infected B-cells appears to be the dominant mechanism of HBV lymphomagenesis at least in the case of diffuse large B-cell lymphoma (DLBCL) [3]. Chronic infection with hepatitis C virus (HCV), a hepatotropic RNA virus, is also associated with increased risk of B-cell NHL particularly of marginal zone (MZ) type [4]. Continual antigenic stimulation by the virus is believed to be the major driver of accumulation of mutations and of the eventual development of lymphomas, a view especially supported by the frequent regression of low-grade NHL after clearance of HCV [5].

A recent meta-analysis of 58 studies reported an average odds ratio (OR) of 2.46 (95% Cl: 1.97–3.07) for B-cell NHL in subjects with surface antigen positive (HBsAg^pos^) HBV infection [2]; concerning NHL subtypes, DLBCL and follicular lymphoma were significantly associated with HBV infection whereas chronic lymphocytic leukemia/small lymphocytic lymphoma was not.

Two studies, both from China where HBV is highly endemic, have addressed the clinical and molecular features of HBV-associated DLBCL, concurring that in patients with chronic HBsAg^pos^ infection, DLBCL occurs earlier in life and has a more aggressive course than in HBsAg^neg^ patients [3, 6]. At the molecular level, HBsAg^pos^ DLBCL had, compared to HBsAg^neg^ DLBCL, an increased total mutation load especially involving BCL6 and the FOXO signaling pathway; this was believed to reflect an infection-driven hyperactive status of B-cells leading to enhanced mutational activity [3]. This study disconfirmed the suggestion raised by the other study [6] that chronic antigenic stimulation by HBsAg could drive DLBCL; it must be noted, however, that chronic antigenic stimulation by HBV could cause other monoclonal B-cell lymphoproliferative disorders such as type 2 mixed cryoglobulinemia vasculitis [7, 8].

Most studies on the association of HBV with increased risk of NHL investigated patients with HBsAg^pos^ chronic infection [2], while fewer studies addressed HBsAg^neg^ patients with past infection defined by the positivity of hepatitis B core antibody (HBcAb^pos^), a life-long serological marker of previous HBV infection. HBsAg^neg^HBcAb^pos^ past infection includes patients with resolved infection or with occult HBV infection (OBI), which is defined as the presence of replication-competent covalently closed circular DNA (cccDNA) of HBV in the blood and/or in the liver of HBsAg^neg^/HBcAb^pos^ subjects [9]. Determining the real prevalence of OBI, which is roughly estimated between 0 and 4.6% of HBsAg^neg^/HBcAb^pos^ subjects by the determination of HBV DNA in blood, is extremely challenging because of methodological limitations, of different target populations studied [9]. More importantly, HBV DNA is detected much more frequently in the liver (62% of HBsAg^neg^/HBcAb^pos^ subjects) than in the blood [10]. Nevertheless, for practical reasons, the HBsAg^neg^HBcAb^pos^ serological status is often used as a surrogate marker to identify OBI in blood/organ donors, in people at risk of reactivation because of immunosuppressive therapies and for epidemiological studies [9]. Studies on the relationship between past HBV infection or OBI and the risk of NHL are scarce and have provided inconsistent results depending at least in part on different endemic areas and study design [11–16]. In this study, we investigated in Italy, a low endemic country for HBsAg, the association of HBcAb^pos^ or HBsAg^neg^/HBcAb^pos^ serological statuses with different B-cell NHL subtypes.

## Methods

### Study cohort

This is a retrospective single-center study involving a cohort of patients with a diagnosis of B-cell NHL seen at the Department of Hematology between 2001 and 2022 (median 2013). We reviewed the available clinical records and extracted 947 cases well classifiable as to B-cell NHL subtype and to HBV serology; patients with HCV or HIV infection or coinfection were excluded from the study. Most patients were lifelong residents in the Lazio region, while 22 were immigrants from high endemic areas (Eastern Europe and Northern Africa); 16 of these patients had indolent B-cell NHLs and 6 had DLBCL.

The lymphoma subtypes studied were clinically aggressive DLBCL and B-cell NHLs with a clinically indolent course; the latter included follicular lymphoma (FL), nodal or extranodal marginal zone lymphoma (MZL), chronic lymphocytic leukemia/small lymphocytic lymphoma (CLL/SLL), low-grade NHL not otherwise specified (low-grade NHL), lymphoplasmacytic lymphoma (LPL), splenic marginal zone lymphoma (SMZL), and mucosa-associated lymphoid tissue NHL (MALT NHL).

Patients were stratified according to lack of any serological evidence for chronic or past HBV infection (HBV^neg^, *n* = 855), chronic infection (HBsAg^pos^, *n* = 24), or past (HBsAg^neg^HBcAb^pos^, *n* = 68) infection.

### Laboratory studies

Patients were tested at the time of diagnosis of NHL. Enzyme-linked immunosorbent assays and molecular assays were routinely used between 2001 and 2022 at the central laboratory of the Policlinico Umberto I/Sapienza University Hospital for testing in serum HBsAg, HBsAb, HBcAb, hepatitis B e antigen (HBeAg), hepatitis B e antibody (HBeAb) and HBV DNA.

### Statistical analysis

The values are expressed as number (%) for categorical variables and median (range) for continuous variables. Univariate comparisons were done by the independent-sample t test or the Mann-Whitney *U* test for continuous variables and the Fisher’s exact test for categorical variables. In multivariate comparisons between the DLBCL and indolent NHL groups, the odds ratio (OR), the corresponding 95% confidence interval (95% CI), and the *p*-value were calculated using an unconditional multiple logistic regression model including as matching variables HBV serological status, gender, and immigrant status as categorical and age at diagnosis as continuous variable. All analyses were performed using the GraphPad Prism version 9.1.2 software. A *p*-value of less than 0.05 was considered significant.

## Results

The main demographic variables and the NHL subtype distribution of the patients enrolled in this study are summarized in Table 1, and univariate analysis of differences in HBsAg^pos^ or HBsAg^neg^HBcAb^pos^ serological statuses in patients with different NHL subtypes is reported in Table 2. The univariate analysis did not reveal associations of either chronic or past HBV infection with any subtype of indolent NHL. We did not replicate the findings of other studies [2] indicating an increased prevalence of HBsAg^pos^ infection in FL; when comparing the prevalence of chronic infection in FL with that in the other indolent NHLs, there were 6/307 cases in the former and 6/387 in the latter group (*p* = 0.77).

**Table 1:** NHL subtype prevalence and gender distribution (number of patients (%)) and age at diagnosis (median (range)) in the study cohort

Group	N of pts	Male gender	Age at diagnosis*
Total	947	456 (48)	62 (20–92)
FL	307 (32)	171 (56)	60 (21–92)
DLBCL	253 (26)	115 (45)	61 (20–91)
MZL*	111 (11)	65 (59)	63 (33–89)
CLL/SLL	86 (9)	35 (41)	63 (31–88)
Low-grade NHL	69 (7)	41 (60)	68 (32–90)
LPL	48 (5)	19 (40)	66 (35–91)
SMZL	48 (5)	31 (65)	62 (31–85)
MALT NHL	25 (2.6)	14 (56)	60 (46–84)

Abbreviations: *CLL/SLL* chronic lymphocytic leukemia/small lymphocytic lymphoma, *low-grade NHL* indolent B-cell NHL not otherwise specified, *LPL* lymphoplasmacytic lymphoma, *MALT NHL* mucosa-associated lymphoid tissue lymphoma, *MZL* marginal zone lymphoma, *SMZL* splenic marginal zone lymphoma

*Including extra-nodal (*n* = 94) and nodal (*n* = 17) MZL

**Table 2:** Univariate analysis of the HBV serological profile (number (%)) of patients with different NHL subtypes. For each subtype, values are compared to those observed in pooled patients with the same serological profile

Group	Pooled patients (%)	DLBCL	FL	MZL	CLL/SLL	Low-grade NHL	LPL	SMZL	MALT NHL
Total	947	253	307	111	86	69	48	48	25
(A). HBV^neg^	855 (90)	212 (24)	280 (32)	103 (12)	85 (10)	59 (7)	47 (5)	46 (5)	23 (3)
(B). HBsAg^pos^	24 (2.5)	12 (48)	6 (24)	1 (4)	0	3 (12)	0	0	2 (8)
(C). HBcAb^pos^	68 (7.2)	29 (38)	21 (28)	7 (9)	1 (1)	7 (9)	1 (1)	2 (3)	0
(A) *vs* (B) (A) *vs* (C)		*p* = 0.008 *p* = 0.002	ns	ns	ns	ns	ns	ns	ns

*ns* not statistically significant

In agreement with several studies, mostly from China [2], the prevalence of HBsAg^pos^ chronic infection was significantly higher (*p* = 0.008) among patients with DLBCL. We could not confirm a younger age of HBsAg^pos^ compared to HBsAg^neg^ DLBCL patients (mean ± SD, 62 ± 31 and 59 ± 15 years, respectively) reported in studies from China [3, 6] probably because of our small sample. Remarkably, the univariate analysis indicated a significantly increased (*p* = 0.002) prevalence of HBsAg^neg^HBcAb^pos^ HBV past infection among DLBCL patients and not in patients with various types of indolent B-cell NHL (Table 2). When comparing by univariate analysis DLBCL (*n* = 253) to pooled indolent NHL (*n* = 694) cases, both HBsAg^+^ active infection (*p* = 0.036) and HBsAg^−^HBcAb^+^ past infection (*p* = 0.0042) were more prevalent in DLBCL. Twenty-six HBsAg^neg^HBcAb^pos^ patients were tested for serum HBV DNA and two (7.7%) were found positive.

Opposite to the case for HBsAg^pos^ DLBCL patients [3, 6], our HBsAg^neg^HBcAb^pos^ patients were older than HBV^neg^ patients (66 ± 11 and 59 ± 15 years, *p* = 0.032 by Mann-Whitney *U* test). In addition, DLBCL patients aged 45 years or less were 0/29 among HBsAg^neg^HBcAb^pos^ and 41/212 (19%) among HBV^neg^ (*p* = 0.0063). An older age of HBsAg^neg^HBcAb^pos^ compared to HBV^neg^ DLBCL patients was also reported in a study from Japan [17].

We matched the HBV serological status and other variables in patients with DLBCL or with indolent B-cell NHLs using an unconditional multiple logistic regression model (Table 3). Both HBsAg^pos^ active infection (OR 2.8, 95% CI 1.2–6.3, *p* = 0.014) and HBsAg^neg^HBcAb^pos^ past infection (OR 2.4, 95% CI 1.5–4.0, *p* = 0.0006) were more prevalent among DLBCL than indolent NHL patients. Of the other matching variables, age at the diagnosis of lymphoma and immigrant status was not associated with DLBCL. To confirm that immigration from high endemic areas was not a confounding factor, we adjusted the logistic regression model by eliminating immigrants from the matched groups; the adjusted model yielded OR 2.29 (CI 1.35–3.9, *p* = 0.0019) for HBsAg^neg^HBcAb^pos^ past infection, OR 1.38 (CI 1.0–1.9, *p* = 0.035) for male gender, and OR 0.99 (CI 0.98–1.0, *p* = 0.09) for age at diagnosis.

**Table 3:** Multivariate analysis of the prevalence of HBsAg^pos^ or HBsAg^neg^HBcAb^pos^ status, gender, age at diagnosis, and immigrant status in patients with DLBCL (*n* = 253) or indolent B-cell NHL (*n* = 694)

Variable	OR	95% CI	*p*-value
HBsAg^pos^	2.78	1.2–6.3	0.014
Male gender	1.37	1–1.9	0.045
Age at diagnosis	0.99	0.9–1	0.54
Immigrant status	0.7	1.2–6.3	0.55
HBcAb^pos^	2.43	1.45–4.0	0.0007
Male gender	1.4	1.0–1.9	0.028
Age at diagnosis	1.0	0.98–1.0	0.064
Immigrant status	0.4	0.1–1.2	0.15

Male gender, a known risk factor for DLBCL [18], could in principle be a confounding factor in the logistic regression model. Against this possibility, univariate analyses did not reveal male/female differences in the prevalence of HBsAg^neg^HBcAb^pos^ and HBsAg^pos^ serologies in the two groups (not shown).

## Discussion

A case-control study from China, where HBV infection is highly endemic, reported an increased risk of B-cell NHL in the HBsAg^neg^HBcAb^pos^ population, with an OR 2.25 (95% CI 1.96–2.57) similar to the OR 2.14 (95% CI 1.88–2.45) observed in the HBsAg^pos^ population [15]; however, lymphoma subtypes were not specified in that study. Another study from China directly compared aggressive with indolent B-cell NHL subtypes and found an increased prevalence of HBsAg^pos^ and, less significantly, of HBsAg^neg^HBcAb^pos^ subjects in the aggressive NHL group [19]. By contrast, two studies, one from Italy [16] and another from Japan [13], reported an increased risk of NHL in the HBsAg^pos^ population but not in the HBsAg^neg^HBcAb^pos^ population; intriguingly, the study from Japan also reported a lack of association with HCV infection although this is an acknowledged risk factor for B-cell NHL [4]. Other studies from different geographic areas have provided contrasting results. For example, the large Epilymph study [12], involving six low endemic European countries, concluded that HBsAg^neg^HBcAb^pos^ past infection increased the risk of unusual lymphoproliferative disorders such as multiple myeloma and CLL/SLL, the latter being reported as non-HBV-related by most studies [2]; even more surprisingly, no association between HBsAg^pos^ chronic infection and increased risk of developing NHL, including DLBCL, was found. Another study in a low-medium endemic population from Israel [14] reported an increased risk of DLBCL in HBsAg^pos^ but not in HBsAg^neg^HBcAb^pos^ subjects.

Differences in the geographic prevalence of HBV infection and in the stratification by lymphoma subtype have been identified as major confounders in studies on the risk of NHL in infected subjects [2]. Indeed, the most significant associations were reported by studies from China, a high endemic country with the largest population of HBV-infected individuals worldwide. Concerning subtype distribution, the association with B-cell NHL is quite solid and most data concur that DLBCL and FL have the strongest association [2]. Our OR 2.68 (95% CI 1.18–6.03) for the association of HBsAg^pos^ chronic infection with DLBCL is close to the OR 2.06 (95% CI 1.48–2.88) and OR 2.45 (95% CI 2.07–2.89) summary estimates for the risk of DLBCL reported, respectively, in two recent meta-analyses [2, 15].

We could not confirm an increased risk of FL in HBsAg^pos^ patients [2]. This could depend on the combined influence of endemic and genetic factors since the literature data were mostly obtained in high endemic Asian areas and especially in China. Indeed, studies from Israel or Europe failed to confirm this association [12, 14], while a large cohort study from Taiwan, an endemic area, reported that chronic HBV infection was significantly associated with DLBCL but not with FL [20].

Our estimate (OR 2.22) of a higher prevalence of past HBV infection in DLBCL is close to the estimate (OR 2.25, 95% CI 1.96–2.57) for the risk of developing unspecified B-cell NHLs in Chinese patients with past HBV infection [15]. Thus, our findings support those from the latter study and further suggest that HBsAg^neg^ past HBV infection, like HBsAg^pos^ chronic infection, specifically increases the risk of DLBCL.

Concerning the possible tumorigenic mechanisms of HBV past infection, an important issue concerns the role for OBI versus fully resolved infection. Data on OBI and lymphomagenesis are extremely scarce. Chen and colleagues [11] investigated serum HBV DNA in HBsAg^neg^ Chinese patients with NHL and, for comparison, in HBsAg^neg^ patients with non-lymphoid tumors and in healthy volunteers. They found a significantly higher prevalence of OBI in patients with DLBCL compared to both control groups; however, the HBcAb status was not investigated in these patients and, therefore, the relative role of resolved rather than occult infection was not addressed. The prevalence of serum HBV DNA positivity among HBsAg^neg^HBcAb^pos^ first-time blood donors ranges from 0.5 to 5.5% in Italy [21, 22]; these figures are somewhat lower than the 7.7% prevalence of OBI observed in the small number (*n* = 26) of HBsAg^neg^HBcAb^pos^ NHL patients tested in our study that, anyway, did not include DLBCL patients. Determining whether the increased risk of NHL in HBsAg^neg^HBcAb^pos^ subjects is associated with OBI or if resolved infection represents per se a risk factor is extremely challenging for methodological issues [9] and, especially, because a reliable detection of OBI may require the testing of liver tissue [10].

The association of past HBV infection with increased risk of DLBCL could be related to OBI; interestingly, in HBsAg^neg^ DLBCL, tumor cells are enriched, compared to other tissues, in selected cccDNA quasispecies carrying specific mutations that could be associated with lymphomagenesis [23]. A non-mutually exclusive alternative is that HBV exploits a hit-and-run mutagenic strategy, consistent with the observation that in HBsAg^pos^ DLBCL, HBV DNA was apparently not integrated in tumor cells which, nevertheless, harbored a distinctive array of mutations of genes involved in lymphomagenesis [3].

HBV-related and HCV-related NHLs reputedly arise through different oncogenic mechanisms, namely mutation-driven [3] and antigen-driven [4], respectively, but nevertheless these infections share a strong association with DLBCL [3, 4]. HCV-related NHLs, including DLBCL, are often associated with mixed cryoglobulinemia, a benign monoclonal B-cell lymphoproliferative disorder; this suggests a stepwise progression from monoclonal cryoglobulinemia to overtly malignant lymphoproliferation [4]. By contrast, HBV more rarely causes mixed cryoglobulinemia and the association with NHL, which was not observed in any of the patients in this study, is rare although it has been reported with low-grade B-cell NHL [24]. This argues against a mechanism of antigen-driven progression in most cases of HBV-associated NHLs. Conversely, it is possible that HCV-associated DLBCL may not arise only through protracted antigenic pressure but rather through direct mutagenesis like it is reputed to be the case for HBV.

HCV can infect and replicate in B-cells [25], and it has been shown that expression of intracellular viral structural proteins very frequently causes lymphomas in mice with disrupted interferon signaling [26]. Also, HCV transgenic mice expressing the full viral genome in B-cells develops DLBCL with high frequency [27]. Interestingly, a “hit and run” mechanism has been proposed also for HCV lymphomagenesis, based on the observation that in vitro infection of B-cells with HCV induces a “mutator phenotype” that causes frequent mutations in immunoglobulin genes and in oncogenes before the virus leaves the cell [28]. This might explain the rare cases of B-cell NHLs occurring late after the clearance of HCV by antiviral therapy [29]. Thus, HBV and HCV might share “hit and run” mutagenesis as an oncogenic mechanism irrespective of active infection.

In conclusion, our results confirm in a low endemic area for HBsAg the data from high endemic areas showing that HBsAg^pos^ chronic HBV infection is associated in with increased risk of DLBCL. We also confirm data from China that also HBsAg^neg^HBcAb^pos^ patients with past HBV infection are at increased risk of developing NHL. More importantly, we provide novel evidence for a specific association of past HBV infection with DLBCL. Whether OBI is necessary for lymphomagenesis associated with past infection remains unanswered. Our findings extend the risk of HBV-associated DLBCL to HBsAg^neg^HBcAb^pos^ people with past infection, a population that among Italian blood donors is 12 to 25-fold larger than the HBsAg^pos^ population [21, 22]. Thus, our results provide clues about both HBV-related lymphomagenesis and public health issues. Our study needs confirmation by studies in areas or populations with different rates of chronic or past HBV infection. Finally, it must be acknowledged that our study has limitations such as the retrospective nature of the study design, to be monocentric, including a relatively small number of patients for several lymphoma subtypes and no information on a possible role for OBI in DLBCL.

## Data Availability

The data supporting this study’s findings are available from the corresponding author, MV, upon reasonable request.
